# Supplementary material for: Heat adaptation of phage T7 under an extended genetic code
Source: Virus Evol. 2021 Dec 1;7(2):veab100. doi: 10.1093/ve/veab100 (PMC8923235; doi:10.1093/ve/veab100)
Supplement: veab100_Supp [file veab100_supp.zip › Sequencing_Analysis.zip!/Sequencing_Analysis/S3/evidence/RA_31.html]

BRESEQ :: Evidence


| Read alignment evidence... | | | | | | | | | | | |
| --- | --- | --- | --- | --- | --- | --- | --- | --- | --- | --- | --- |
|  | seq id | position | | ref | new | freq | score (cons/poly) | reads | annotation | genes | product |
| \* | NC\_001604.1 | 28,692 | 0 | A | G | 36.2% | 1098.1 / inf | 1448 | intergenic (–/–) | *–/–* | –/– |
| Reads supporting (aligned to +/- strand):  **ref** base A (460/453);  **new** base G (264/254);  **total** (734/714) | | | | | | | | | | | |
| Fisher's exact test for biased strand distribution *p*-value = 8.69e-01 | | | | | | | | | | | |
| Kolmogorov-Smirnov test that lower quality scores support variant *p*-value = 7.58e-01 | | | | | | | | | | | |

Only 100 of 1505 total aligned reads displayed.  

`AGGTATGGGCTATCGTGCAGCAACCACTCAGGCCGAACAGCCAAGGTCAAGCCTATTGGACACCATTGGTCGGTTCGCTAAGGCTGGTGCCGATATGTATACCGCTAAGGAACAACGAGCACGAGACCTAGCTGATGAACGCTCTAACGAGATTATCCGTAAGCTGACCCCTGAGCAACGTCGAGAAGCTCTCAACAACGGGACCCTTCTGTATCAGGATGACCCATACGCTATGGAAGCACTCCGAGTCAAGACTGGTCGTAACGCTGCGTATCTTGTGGACGATGACGTTATGCAGAAGATAAAAGAGGGTGTCTTCCGTACTCGCGAAGAGATGGAAGAGTATCGCCATAGTCGCCTTCAAGAGGGCGCTAAGGTATACGCTGAGCAGTTCGGCATCGACCCTGAGGACGTTGATTATCAGCGTGGTTTCAACGGGGACATTACCGAGCGTAACATCTCGCTGTATGGTGCGCATGATAACTTCTTGAGCCAGCAAGCTCAGAAGGGCGCTATCATGAACAGCCGAGTGGAACTCAACGGTGTCCTTCAAGACCCTGATATGCTGCGTCGTCCAGACTCTGCTGACTTCTTTGAGAA  >  NC_001604.1/28393‑28992`  

                                                                                                                                                                                                                                                                                                           `|`                                                                                                                                                                                                                                                                                                              
`aGGTATGGGCTATCGTGCAGCAACCACTCAGGCCGAACAGCCAAGGTCAAGCCTATTGGACACCATTGGTCGGTTCGCTAAGGCTGGTGCCGATATGTATACCGCTAAGGAACAACGAGCACGAGACCTAGCTGATGAACGCTCTAACGAGATTATCCGTAAGCTGACCCCTGAGCAACGTCGAGAAGCTCTCAACAACGGGACCCTTCTGTATCAGGATGACCCATACGCTATGGAAGCACTCCGAGTCAAGACTGGTCGTAACGCTGCGTATCTTGTGGACGATGACGTTATGcagcag                                                                                                                                                                                                                                                                                                             <  1:100013/301‑1 (MQ=255)`  
      `gggCTATCGTGCAGCAACCACTCAGGCCGAACAGCCAAGGTCAAGCCTATTGGACACCATTGGTCGGTTCGCTAAGGCTGGTGCCGATATGAATACCGCTAAGGAACAACGAGCACGAGACCTAGCTGATGAACGCTCTAACGAGATTATCCGTAAGCTGACCCCTGAGCAACGTCGAGAAGCTCTCAACAACGGGACCCTTCTGTATCAGGATGATCCATACGCTATGGAAGCACTCCGAGTCAAGACTGGTCGTAAAGCTGCGTATCTTGTGGACGATGACGTTATGCAGCAGCTtaaa                                                                                                                                                                                                                                                                                                       >  1:71391/1‑301 (MQ=255)`  
       `gtataagagaccacaACCAATCAGGCCGAACAGCCAAGGTCAAGCCTATTGGACAACATTGGTCCGTTCGCTAAAGCTGGTGCCGATATGTATACCGCTAAGGAACAACGAGCACGAGACCTAGCTGATAAACGCTCAAACGGGATTATCCGTAAGCTGATCCCTGAGCAACGTCGAAAAGCTCTCAACAACGGGACCCTTCTGTATCAGGATGACCCATACGCTATGGAAGCACTCCGAGTCAAGACTGGTCGTAACGCTGCGTATCTTGTGGACGATGACGTTATGCAGAGGATAAAag                                                                                                                                                                                                                                                                                                      <  1:315149/288‑1 (MQ=255)`  
       `ggCTATCGTGCAGCAACCACTCAGGCCGAACAGCCAATGTCAAGCCTATTGTACCCCATTGGTCTGTTCTATAAGTCTGGTGCCGATATATATACCGCTAAGGAACAACTAGAACGAGACCTAGCTGATGAACCCTCTAACGAGATTATCCGTAATCTGACCCCTTAGCAACGTCGAGAAGCTCTCAACAACGGGACCCTTCTGTATCAGGACGCCCCATACGCTATGGAAGTACTCAAAGTCAAGACTTGTCGTAACGCTGCGTATCTTGTGGACGATGACGTTATTCAGTGGATAAAag                                                                                                                                                                                                                                                                                                      <  1:157728/301‑1 (MQ=255)`  
       `ggCTATCGTGCAGCAACCACTCAGGCCGAACAGCCAAGGTCAAGCCTATTGGACACCATTGGTCGGTTCGCTAAGGCTGGTGCCGATATGTATACCGCTAAGGAACAACGAGCACGAGACCTAGCTGATGAACGCTCTAACGAGATTATCCGTAAGCTGACCCCTGAGCAACGTCGAGAAGCTCTCAACAACGGGACCCTTCTGTATCAGGATGACCCATACGCTATGGAAGCACTCCGAGTCAAGACTGGTCGTAACGCTGCGTATCTTGTGGACGATGACGTTATGCAGCAGATAAAag                                                                                                                                                                                                                                                                                                      <  1:104577/301‑1 (MQ=255)`  
       `ggCGATCGTGCACAAACCACTCAGGCCGAACAGCCAAGGTCAAGCCGATTGGACACCATTGGTCGGTTCGCTAAGGCTGGTGCCGATATGTATACCGCTAAGGAACAACGAGCACGAGACCTAGCTGATGAACGCTCTAACGAGATTATCCGTAAGCTGACCCCTGAGCAACGTCGAGAAGCTCTCAACAACGGGACCCTTCTGTATCAGGATGACCCATACGCTATGGAAGCACTCCGAGTCAAGACTGGTCGTAACGCTGCGTATCTTGTGGACGATGACGTTATGCAGAGGATAAAag                                                                                                                                                                                                                                                                                                      <  1:336863/301‑1 (MQ=255)`  
       `caagaTCGTGCAGCAACCACTCAGGCCGAACAGCCAAGGTCAAGCCTATTGGACACCAGTGGTCGGTTCGCTAAGGCTGGTGCCGATATGTATACCGCTAAGGAACAACGAGCACGAGACCTAGCTGATGAACGCTCTAACGAGATTATCCGTAAGCTGACCCCTGAGCAACGTCGAGAAGGTCTCAACAACGGGACCCTTCTGTATCAGGATGACCCATACGCTATGGAAGCACTCCGAGTCAAGACTGGTCGTAACGCTGCGTATCTTGTGGACGATGACGTTATGCAGCAGATAAAag                                                                                                                                                                                                                                                                                                      <  1:1391/297‑1 (MQ=255)`  
         `gtATCGTGCAGCAACCACTCAGGCCAAACAGCCAAGGTCAAGCCTATTGGACACCATTGGTCGGTTCGCTAAGGCTGGTGCCGATATGTATACCGCTAAGGAACAACGAGCACGAGACCTAGCTGATGAACGCTCTAACGAGATTATCCGTAAGCTGACCCCTGAGCAACGTCGAGAAGCTCTCAACAACGGGACCCTTCTGTATCAGGATGACCCATACGCTATGGAAGCACTCCGAGTCAAGACTGGTCGTAACGCTGCGTATCTTGTGGACGATGACGTTATGCAGCAGATAAAagag                                                                                                                                                                                                                                                                                                    <  1:249936/300‑1 (MQ=255)`  
           `aTCGTGCAGCAACCACTCAGGCCGAACAGCCAAGGTCAAGCCTATTGGACACCATTGGTCGGTTCGCTAAGGCTGGTGCCGATATGTATACCGCTAAGGAACAACGAGCACGAGACCTAGCTGATGAACGCTCTAACGAGATTATCCGTAAGCTGACCCCTGAGCAACGTCGAGAAGCTCTCAACAACGGGACCCTTCTGTATCAGGATGACCCATACGCTATGGAAGCACTCCGAGTCAAGACTGGTCGTAACGCTGCGTATCTTGTGGACGATGACGTTATGCAGAGGATAAACGAggg                                                                                                                                                                                                                                                                                                  >  1:260810/1‑301 (MQ=255)`  
           `aTCGTGCAGCAACCACTCAGGCCGAACAGCCAAGGTCAAGCCTATTGGACACCATTGGTCGGTTCGCTAAGGCTGGTGCCGATATGTATACCGCTAAGGAACAACGAGCACGAGACCTAGCTGATGAACGCTCTAACGAGATTATCCGTAAGCTGACCCCTGAGCAACGTCGAGAAGCTCTCAACAACGGGACCCTTCTGTATCAGGATGACCCATACGCTATGGAAGCACTCCGAGTCAAGACTGGTCGTAACGCTGCGTATCATGTGGACGATGACGTTATGCAGCAGCTAAAagagct                                                                                                                                                                                                                                                                                                  >  1:211019/1‑299 (MQ=255)`  
           `aTCGTGCAGCAACCACTCAGGCCGAACAGCCAAGGTCAAGCCTATTGGACACCATTGGTCGGTTCGCTAAGGCTGGTGCCGATATGTATACCGCTAAGGAACAACGAGCACGAGACCTAGCTGATGAACGCTCTAACGAGATTATCCGTAAGCTGACCCCTGAGCAACGTCGAGAAGCTCTCAACAACGGGACCCTTCTGTATCAGGATGACCCATACGCTATGGAAGCACTCCGAGTCAAGAATGGTCGTAACGCTGCGTAGCTTGTGGACGATGACGTTCTGCAGCAGATAAAagagct                                                                                                                                                                                                                                                                                                  >  1:94978/1‑299 (MQ=255)`  
             `cgggacacaACCACTCGGGTCGAACAGCCAAGGTCAAGGCTATTGGACACCATTGGGCGGTTTGCTAAGGCTGGTGCCGATATGTATACCGGTAAGGAACAACGAGCACGAGACCTAGCTTATGAACGCTCTAACGAGATTATCCGTAAGCTGACCCCTGAGCAACGTCGAGAAGCTCTCAACAACGGGACCCTTCTGTATCAGGATGACCCATACGCTATGGAAGCACTCCGAGTCAAGACTGGTCGTAACGCTGCGTATCTTGTGGACGATGACGTTATGCAGAGGATAAAAGAGGgtg                                                                                                                                                                                                                                                                                                <  1:323334/294‑1 (MQ=255)`  
                            `tcggagatgtgtataagagacagacTATTGGACACCATTGGTCGGTTCGCTAAGGATGGTGCCGATATGTATACCGATAAGGAACAACGAGCACGAGACCTAGCTGATGAACGCTCTAACGAGATTATCCGTAAGCTGACCCCTGAGCAACGTCGAGAAGCTCTCAACAACGGGACCCTTCTGTATCAGGATGACCCATACGCTATGGAAGCACTCCGAGTCAAGACTGGTCGTAACGCTGCGTATCTTGTGGACGATGACGTTATGCAGCAGATAAAAGAGGGTGTCTTCCGTACTcgcg                                                                                                                                                                                                                                                                                 <  1:246270/277‑1 (MQ=255)`  
                               `gCCGAACAGCCAAGGTCAAGCCTATTGGACACCATTGGTCGGTTCGCTAAGGCTGGTGCCGATATGTATACCGCTAAGGAACAACGAGCACGAGACCTAGCTGAAGAACGCTCTAACGAGATTATCCGTAAGCTGACCCCTGAGCAACGTCGAGAAGCTCTCAACAACGGGACCCTTCTGTATCAGGATGACCCATACGCTATGGAAGCACTCCGAGTCAAGACTGGTCGTAACGCTGCGTATCTTGTGGACGATGACGTTATGCAGCAGATAAAAGAGGGTGTCTTCCGTACTCGCGAag                                                                                                                                                                                                                                                                              <  1:3465/301‑1 (MQ=255)`  
                                          `aaGGGCAAGCCTAGTGGACAGCATTGGTCGGGTCGATAAGGCTGGTGCCGATATGTATACCGCTAAGGAACAACGAGAACGAGACCTAGCTGATGAACGATCTAACGAGATTATCCGTAAGCTGACCCCTGAGCAACGTCGAGAAGCTCTCAACAACGGGACCCTTCTGTATCAGGATGACCCATACGCTATGGAAGCACTCCGAGTTAAGACTGGTCGTAACGCTGCGTATCTTGTGGACGATGACGTTATGCAGCAGATAAAAGAGGGTGTCTTCCGTACTCGAGAAGAGATGGAagag                                                                                                                                                                                                                                                                   <  1:150391/301‑1 (MQ=255)`  
                                            `ggTCAAGCCTATTGGACACCATTGGTCGGTTCGCTAAGGCTGGTGCCGATATGTATACCGCTAAGGAACAACGAGCACGAGACCTAGCTGATGAACGCTCTAACGAGATTATCCGTAAGCTGACCCCTGAGCAACGTCGAGAAGCTCTCAACAACGGGACCCTTCTGTATCAGGATGACCCATACGCTATGGAAGCACTCCGAGTCAAGACTGGTCGTAACGCTGCGTATCTTGTGGACGATGACGTTATGCAGAGGATAAAAGAGGGTGTCTTcctgtctcttatacacatctccggggc                                                                                                                                                                                                                                                                 >  1:220231/1‑276 (MQ=255)`  
                                             `ctCAATCCAATTGGACACCATTGGTCGGTTCGCTAAGGCTGGTTCCGATATGTATACCGCTAAGGAACAACGAGCACGAGACCTAGCGTATGAACGCTCTAACGAGATTATCCGTAAGCTGACACCTGAGCAACGTCGAGAAGCTCTCAACAACGGGACCCTTCTGTATCAGGATGACCCATACGCTATGGAAGCACTCCGAGTCAAGACTGGTCGTAACGCTGCGTATCTTGTGGACGATGACGTTATGCAGAGGATAAAAGAGGGTGTCTTACGTACTCGCGAAGAGATGGAAGAGTAt                                                                                                                                                                                                                                                                <  1:92071/300‑1 (MQ=255)`  
                                                  `gagacagggACACCATTGGTCGGTTCGCTAAGGCTGGTGCCGATATGTATACCGCTAAGGAACAACGAGCACGAGACCTAGCTGATGAACGCTCTAACGAGATTATCCGTAAGCTGACCCCTGAGCAACGTCGAGAAGCTCTCAACAACGGGACCCTTCTGTATCAGGATGACCCATACGCTATGGAAGCACTCCGAGTCAAGACTGGTCGTAACGCTGCGTATCTTGTGGACGATGACGTTATGCAGAGGATAAAAGAGGGTGTCTTCCGTACTCGCGAAGAGATGGAAGAGTATCGCCa                                                                                                                                                                                                                                                           <  1:214089/294‑1 (MQ=255)`  
                                                  `acagaTTGGACACCATTGGTCGGTTCGCTTAGGCTGGTGCCGATATGTATACCGCTAAGGAACAACGAGCACGAGACCTAGCTGATGAACGCTCTAACGAGATTATCCGTAAGCTGACCCCTGAGCAACGTCGAGAAGCTCTCAACAACGGGACCCTTCTGTATCAGGATGACCCATACGCTATGGAAGCACTCCGAGTCAAGACTGGTCGTAACGCTGCGTATCTTGTGGACGATGACGTTATGCAGAGGATAAAAGAGGGTGTCTTCCGTACTCGCGAAGAGATGGAAGAGTATCGCCa                                                                                                                                                                                                                                                           <  1:226856/297‑1 (MQ=255)`  
                                                    `cTATTGGACACCATTGTTCGGTTCGCTAAGGCTGGTGCCGATATGTATACCGCTAAGGTACAACGAGCACGAGACCTAGCTGATGAACGCTCTAACGAGATTATCCGTAAGCTGACCCCTGAGCAACGTCGAGAAGCTCTCAACAACGGGACCCTTCTGTATCAGGATGACCCATACGCTATGTAAGCACTCCGAGTCAAGACTGGTCGTAACGCTGCGTATCTTGTGGACGATGACGTTATGCAGAGGATAAAAGAGGGTGTCTTCCGTACTCGCGAAGAGATGGAAGAGTATCGCCATa                                                                                                                                                                                                                                                         >  1:122801/1‑301 (MQ=255)`  
                                                    `cTATTGGACACCATTGGTCGGTTCGCTAAGGCTGGTGCCGATATGTATACCGCTAAGGAACAACGAGCACGAGACCTAGCTGATGAACGCTCTAACGAGATTATCCGTAAGCTGACCCCTGAGCAACGTCGAGAAGCTCTCAACAACGGGACCCTTCTGTATCAGGATGACCCATACGCTATGGAAGCACTCCGAGTCAAGACTGGTCGTAACGCTGCGTATCTTGTGGACGATGACGTTATGCAGCCGATAAAAGAGGGTGTCTTCCGTAcctgtctcttatacacatccccgaccccac                                                                                                                                                                                                                                                         >  1:285859/1‑272 (MQ=255)`  
                                                      `aTTGGACACCATTGGTCGGTTCGCTAAGGCTGGTGCCGATATGTATACCGCTAAGGAACAACGAGCACGAGACCTAGCTGATGAACGCTCTAACGAGATTATCCGTAAGCTGACCCCTGAGCAACGTCGAGAAGCTCTCAACAACGGGACCCTTCTGTATCAGGATGACCCATACGCTATGGAAGCACTCCGAGTCAAGACTGGTCGTAACGCTGCGTATCTTGTGGACGATGACGTTATGCAGAGGATAAAAGAGGGTGTCTTCCGTACTCGCCAAGCGATGGCAGAGTATCGCCATAGt                                                                                                                                                                                                                                                       >  1:233479/1‑301 (MQ=255)`  
                                                        `tgatcacCATAGGGGGGTGCGCAAAGGCAGGTGCCGATATGTAAACCGCTAAGGAACAACGAGCACGAGACCTAGCTGATGAACGAGCAAACGAGATTATACGTAAGCTGACCCCTGAGCAACGTCGAGAAGCTCTCAACAACGGGACCCTTCTGTATCAGGATGACCCATACGCTAAGGAAGCACTCCGAGTCAAGACTGGTCGTAACGCTGCGTATCTTGTGGACGATGACGTTATGCAGCAGATAAAAGAGGGTGTCTTCCGTACTCGCGAAGAGATGGAAGAGTATCGCCATAGTCg                                                                                                                                                                                                                                                     <  1:229296/297‑1 (MQ=255)`  
                                                         `ggACACCATTGGTCGGTTCGCTAAGGCTGGTGCCGATATGTATACCGCTAAGGAACAACGAGCACGAGACCTAGCTGATGAACGCTCTAACGAGATTATCCGTAAGCTGACCCCTGAGCAACGTCGAGAAGCTCTCACCAACGGGACCCTTCTGTATCAGGATGACCCATACGCTATGGAAGCACTCCGAGTCAAGACTGGTCGTAACGCTGCGTATCTTGTGGACGATGACGTTATGCAGCAGATAAAAGAGGGTGTCTTCCGTACTCGCGAAGAGATGGAAGAGTATCGCCATAGTCGc                                                                                                                                                                                                                                                    >  1:148090/1‑301 (MQ=255)`  
                                                              `ccATTGGTCGGTTCGCTAAGGCTGGTGCCGATATGTATACCGCTAAGGAACAACGAGCACGAGACCTAGCTGATGAACGCTCTAACGAGATTATCCGTAAGCTGACCCCTGAGCAACGTCGAGAAGCTCTCAACAACGGGACCCTTCTGTATCAGGATGACCCATACGCTATGGAAGCACTCCGAGTCAAGACTGGTCGTAACGCTGCGTATCTTGTGGACGATGACGTTATGCAGCAGATAAAAGAGGGTGTCTTCCGTACTCGCGAAGAGATGGAAGAGTATCGCCATAGTCGCCTTCa                                                                                                                                                                                                                                               >  1:282269/1‑301 (MQ=255)`  
                                                              `ccATTGGTCGGTTCGCTAAGGCTGGTGCCGATATGTATACCGCTAAGGAACAACGAGCACGAGACCTAGCTGATGAACGCTCTAACGAGATTATCCGTAAGCTGACCCCTGAGCAACGTCGAGAAGCTCGCAACAACGGGACCCTTCTGTATCAGGATGACCCATACGCTATGGAAGCACTCCGAGTCAAGACTGGTCGTAACGCTGCGTATCTTGTGGACGATGACGTTATGCAGAGGATAAAAGAGGGTGTCTTCCGTACTCTCGAAGCGATGGAAGAGTATCGCCATAGTCGCCTTCa                                                                                                                                                                                                                                               >  1:153332/1‑301 (MQ=255)`  
                                                                 `ttggttggTTCGCTAAGGCTGGTGCCGATATGTATACCGCTAAGGAACAACGAGCACGAGACCTAGCTGATGAACGCTCTAACGAGATTATCCGTAAGCTGACCCCTGAGCAACGTCGAGAAGCTCTCAACAACGGGACCCTTCTGTATCAGGATGACCCATACGCTATGGAAGCACTCCGAGTCAAGACTGGTCGTAACGCTGCGTATCTTGTGGACGATGACGTTATGCAGCAGATAAAAGAGGGTGTCTTCCGTACTCGCGAAGAGATGGAAGAGTATCGCCATAGTCGCCTTCAaga                                                                                                                                                                                                                                            <  1:174088/301‑1 (MQ=255)`  
                                                                           `cGCTAAGGCTGGTGCCGATATGTATACCGCTAAGGAACAACGAGCACGAGACCTAGCTGATGAACGCTCTAACGAGATTATCCGTAAGCTGACCCCTGAGCAACGTCGAGAAGCTCTCAACAACGGGACCCTTCTGTATCAGGATGACCCATACGCTATGGAAGCACGCCGAGTCAAGACTGGTCGTAACGCTGCGTATCTTGTGGACGATGACGTTATGCAGAGGAGAAAAGAGGGTGTCTGCCGTACTCGCGAAGAGATGGAAGAGTATCGCCATAGTCGCCTTCAAGAGGGCGCTAAg                                                                                                                                                                                                                                  >  1:206810/1‑301 (MQ=255)`  
                                                                                 `aaagagacaggATATGTATACCGCTAAGGAACAACGAGCACGAGACCTAGCTGATGAACGCTCTAACGAGATTATCCGTAAGCTGACCCCTGAGCAACGTCGAGAAGCTCTCAACAACGGGACCCTTCTGTATCAGGATGACCCATACGCTATGGAAGCACTCCGAGTCAAGACTGGTCGTAACGCTGCGTATCTTGTGGACGATGACGTTATGCAGAAGATAAAAGAGGGTGTCTTCCGTACTCGCGAAGAGATGGAAGAGTATCGCCATAGTCGCCTTCAAGAGGGCGCTAAGGTATAc                                                                                                                                                                                                                            <  1:96813/291‑1 (MQ=255)`  
                                                                                   `taagagacagtatGTATAACGCTAAGGAACAACGAGCACGAGACCTAGCTGATGCACGCTCTAACGAGATTATCCGTAAGCTGACCCCTGAGCAACGTCGAGAAGCTCTCAACAACGGGACCCTTGTGTATCAGGATGACCCATACGGTATGGAAGCACTACGAGTCAAGACTGGTCGTAACGCTGCGTATCTTGTGGACGAAGACGTTATGCAGCAGATAACAGAGGGTGTCTTCCGTACTCGCGAAGAAATGGAAGAGTATCGCCATAGTCGCCTTCAAGAGGGCGCTAAGGTATACGc                                                                                                                                                                                                                          <  1:54327/291‑1 (MQ=255)`  
                                                                                        `gCCGATATGTATACCGCTAAGGAACAACGAGCACGAGACCTAGCTGATGAACGCTCTAACGAGATTATCCGTAAGCTGACCCCTGAGCAACGTCGAGAAGCTCTCAACAACGGGACCCTTCTGTATCAGGATGACCCATACGCTATGGAAGCACTCCGAGTCAAGACTGGTCGTAACGCTGCGTATCTTGTGGACGATGACGTTATGCAGAAGATAAAAGAGGGTGTCTTCCGTACTCGCGAAGAGATGGAAGAGTATCGCCATAGTGGCATTCAAGAGGGCGCTAAGGTATACGCTGAgg                                                                                                                                                                                                                     >  1:192947/1‑300 (MQ=255)`  
                                                                                           `gATATGTAGACCGCTAAGGAACAACGAGCACGAGACCTAGCTGACGAACGCTCTAACGAGATTATCCGTAAGCTGACCCCTGAGCAACGTCGAGAAGCTCTCAACAACGGGACCCTTCTGTATCAGGATGACCCATACGCTATGGAATCACTCCGAGTCAAGACTGGTCGTAACGCTGCGTATCTTGTGGACGATGACGTTATGCAGAAGATAAAAGAGGGTGTCTTCCGTACTCGCGAAGAGATGGAAGAGTATCGCCATAGTCGCCTTCAAGAGGGCGCTAAGGTATACGCGGAGCAGt                                                                                                                                                                                                                  >  1:171367/1‑301 (MQ=255)`  
                                                                                             `tatGTATACCGCTAAGGAACAACGAGCACGAGACCTAGCTGATGAACGCTCTAACGAGATTATCCGTAAGCTGACCCCTGAGCAACGTCGAGAAGCTCTCAACAACGGGACCCTTCTGTATCAGGATGACCCATACGCTATGGAAGCACTCCGAGTCAAGACTGGTCGTAACGCTGCGTATCTTGTGGACGATGACGTTATGCAGCAGATAAAAGAGGGTGTCTTCCGTACTCGCGAAGAGATGGAAGAGTATCGCCATAGTCGCCTTCAAGCGGGCGCTAAGGTATACGcctgtctcttt                                                                                                                                                                                                                >  1:275304/1‑291 (MQ=255)`  
                                                                                                `gTATACCGCTAAGGAACAACGAGCACGAGACCTAGCTGATGAACGCTCTAACGAGATTATCCGTAAGCTGACCCCTGAGCAACGTCGAGAAGCTCTCAACAACGGGACCCTTCTGTATCAGGATGACCCATACGCTATGGAAGCACTCCGAGTCAAGACTGGTCGTAACGCTGCGTATCTTGTGGACGATGACGTTATGCAGAGGATAAAAGAGGGTGTCTTCCGTACTCGCGAAGAGATGGAAGAGTATCGCCATAGTAGCCTTCTAGAGGGCGCTAAGGTATACGCTGAGCAgctgttt                                                                                                                                                                                                             >  1:166589/1‑295 (MQ=255)`  
                                                                                                `gTATACCGCTAAGGAACAACGAGCACGAGACCTAGCTGATGAACGCTCTAACGAGATTATCCGTAAGCTGACCCCTGAGCAACGTCGAGAAGCTCTCAACAACGGGACCCTTCTGTATCAGGATGACCCATACGCTATGGAAGCACTCCGAGTCAAGACTGGTCGTAACGCTGCGTATCTTGTGGACGATGACGTTATGCAGAAGATAAAAGAGGGTGTCTTCCGTAATCGCGAAGAGATGGAAGAGTATCACACTCGTCGACTTCAAGGAGGCGGTAAGGTGTGCGCTGAGGAGTTCGGc                                                                                                                                                                                                             >  1:316860/1‑301 (MQ=255)`  
                                                                                                  `ataCCGCTAAGGAACAACGAGCACGAGACCTAGCTGATGAACGCTCTAACGAGATTATCCGTAAGCTGACCCCTGAGCAACGTCGAGAAGCTCTCAACAACGGGACCCTTCTGTATCAGGATGACCCATACGCTATGGAAGCACTCCGAGTCAAGACTGGTCGTAACGCTGCGTATCTTGTGGACGATGACGTTATGCAGAGGATAAAAGAGGGTGTCTTCCGTACTCGCGAAGAGATGGAAGAGTATCTCCATAGTCGCCTTCAAGAGGGCGCTAAGGTATACGCTGAGCAGTTCGGCAt                                                                                                                                                                                                           <  1:76285/301‑1 (MQ=255)`  
                                                                                                        `cTAAGGAACAACGAGCACGAGACCTAGCTGATGAACGCTCTAACGAGATTATCCGTAAGCTGACCCCTGAGCAACGTCGAGAAGCTCTCAACAACGGGACCCTTCTGTATCAGGATGACCCATACGCTATGGAAGCACTCCGAGTCAAGACTGGTCGTAACGCTGCGTATCTTGTGGACGATGACGTTATGCAGCAGATAAAAGAGGGTGTCTTCCGTACTCGCGACGAGATGGAAGAGTATCGCCATAGTCGCCTTCAAGAGGGCGCTAAGGTATACGCTGAGCAGTTCGGCATCGAccc                                                                                                                                                                                                     >  1:129794/1‑301 (MQ=255)`  
                                                                                                                   `cGAGCACGAGACCTAGCTGATGAACGCTCTAACGAGATTATCCGTAAGCTGACCCCGGAGCAACGTCGAGAAGCTCTCAACAACGGGACCCTTCTGTATCAGGATGACCCATACGCTATGGAAGCACTCCGAGTCAAGACTGGTCGTAACGCTGCGTATCTTGTGGACGATGACGTTATGCAGAGGATAAAAGAGGGTGTCTTCCGTACTCGCGAAGAGATGGAAGAGTATCGCCATAGTCGCCTTCAAGAGGGCGCTAAGGTATACGCTGAGCAGTTCGGCATCGACCCTGAGGACGTTg                                                                                                                                                                                          <  1:28990/301‑1 (MQ=255)`  
                                                                                                                       `aaCGAGACCTAGCTGATGAACGCTCTAACGAGATTATCCGTAAGCTGACCCCTGAGCAACGTCGAGAAGCTCTCAACAACGGGACCCTTCTGTATCAGGATGACCCATACGCTATGGAAGCACTCCGAGTCAAGACTGGTCGTAACGCTGCGTATCTTGTGGACGATGACGTTATGCAGCAGATAAAAGAGGGTGTCTTCCGTACTCGCGAAGAGATGGAAGAGTATCGCCATAGCCGCCTTCAAGAGGGCGCTAAGGTATACGCTGAGCAGTTCGGCATCGACCCTGAGGACGTTGATTa                                                                                                                                                                                      <  1:35903/300‑1 (MQ=255)`  
                                                                                                                         `cGAGACCTAGCTGATGAACGCTCTAACGAGATTATCCGTAAGCTGACCCCTGAGCAACGTCGAGAAGCTCTCAACAACGGGACCCGTCTGTATCAGGATGACCCATACGCTATGGAAGCACTCCGAGTCAAGACTGGTCGTAACGCTGCGTATCTTGTGGACGATGACGTTATGCAGAGGATAAAAGAGGGTGTCTTCCGTACTCGCGAAGAGATGGAAGAGTATCGCCATAGTCGCCTTCAAGAGGGCGCTAAGGTATACGCTGAGCAGTTCGGCATCGACCCTGAGGACGTTGATTAtg                                                                                                                                                                                    <  1:132577/301‑2 (MQ=255)`  
                                                                                                                           `agaCCTAGCTGATGAACGCTCGAACGGGATTATCCGTAAGCTGAACCCTGAGCAACGTCGAGACGCTCTCAACAACGGGACCCTTCTGTATCAGGATGACCCATCCGCTATGGAAGCACTCCGAGTCAAGACTGGTCGTAACGCTGTGTATTTTGTGGACGATGACGTTATGCAGCAGATAAAAGAGGATGTCTTCCGTACTCGCGAAGAGATGGAAGAGTATCGCCATAGTCGCCTTCAAGAGGGCGATAAGGTATACGCTGAGCAGTTCGGCATCGACCCTGAGGACGTTGATTATCAg                                                                                                                                                                                  <  1:79522/301‑1 (MQ=255)`  
                                                                                                                                            `gCTCTAACGAGATTATCCGTAAGCTGACCCCTGAGCAACGTCGAGAAGCTCTCAACAACGGGACCCTTCTGTATCCGGATGACCCATATGCTATGGAAGCCCTCCGAGTCAAGACTGGTCGTAACGCTTCGTATCTTGTGGTCGATGTCGCTATGCAGCAGATAAAAGAGGGTGTCTTCCGTACTCGCGACGTGATGGAGGAGTTTCGCCCTTGTCGCCTT‑AAGAGGGAGCTAAGGTATACGCTCAGCAGTTCGGCATCGACCCTGAGGACGTTGATGATCCGCGTGGTTTCCAcctgtct                                                                                                                                                                >  1:40714/1‑295 (MQ=255)`  
                                                                                                                                            `cctctAAAGGGATCAGCCGGAACCTGAGCCTTGATCAACGGTGAGAAGTACTCAACAACGGGAGCCTTCCGTATCGGGATGCCCAATACGGTATGGAAGCACTGCGAGTCAAGGGTGGTCGTAACGCTGCGTATCTTGTGGACGATGACGTTATGCAGAAGATAAAAGAGGGTGTCTGCCGTACTCGCGAAGAGATGGAAGAGTATCGCCATAGTCGCCTGCAAGAGGGCGCTAAGGTATACGCTGAGGAGTTCGGCATCGACCCTGAGGACGTTGATTATCAGCGTGGTTTCAACGGGGa                                                                                                                                                                 <  1:241182/300‑1 (MQ=255)`  
                                                                                                                                             `aagagaCGGGATTATCCGTAAGCTGCCCCCGGAGCAACGTCGAGAAGCTCTCAACAACGGGACCCTTCTGTATCAGGATGACCCATACGCTATGGAAGCACTCCGAGTCAAGACTGGTCGTAACGCTGCGTATCTTGTGGACGATGACGTTATGCAGCAGATAAAAGAGGGTGTCTTCCGTACTCGCGAAGAGATGGAAGAGTATCGCCATAGTCGCCTTCAAGAGGGCGCTAAGGTATACGCTGAGCAGTTCGGCATCGACCCTGAGGACGTTGATTATCAGCGTGGTTTCAACGGGGAc                                                                                                                                                                <  1:180323/296‑1 (MQ=255)`  
                                                                                                                                                    `gagaTTATCCGTAAGCTGACCCCTGAGCAACGTCGAGAAGCTCTCAACAACGGGACCCTTCTGTATCAGGATGACCCATACGCTATGGAAGCACTCCGAGTCAAGACTGGTCGTAACGCTCCGTATCTTGTGGACGATGACGTTATGCAGAGGATAAAAGAGGGTGTCTTCCGTACTCGCGAAGAGATGGAAGAGTATCGCCATAGTCGCCTTCAAGAGGGCGCTAAGGTATACGCTGAGCAGTTCGGCATCGACCCTGAGGACGTTGATTATCAGCGTGGTTTCAACGGGGACATTACCg                                                                                                                                                         <  1:169904/301‑1 (MQ=255)`  
                                                                                                                                                      `gacagtCCGTAAGCTGACCCCTGAGCAACGTCGAGAAGCTCTCAACAACGGGACCCTTCTGTATCAGGATGACCCATACGCTATGGAAGCACTCCGAGTCAAGACTGGTCGTAACGCTGCGTATCTTGTGGACGATGACGTTATGCAGAGGATAAAAGAGGGTGTCTTCCGTACTCGCGAAGAGATGGAAGAGTATCGCCATAGTCGCCTTCAAGAGGGCGCTAAGGTATACGCTGAGAAGTTCGGCATCGACCCTGAGGACGTTGATTATCAGCGTGGTTTCAACGGGGACATTACCGAg                                                                                                                                                       <  1:89437/296‑1 (MQ=255)`  
                                                                                                                                                        `ttATCCGTAAGCTGACCCCTGGGCAACGTCGAGAAGCTCTCAACAACGGGACCCTTCTGTATCAGGATGACCCATTCGCTATGGAAGCACTCCGAGTCCAGACTGGTCTTAACGCTGCGTATCTTGTGGACGATGACGTTATGCAGAGGATAAAAGAGGGTGTCTTCCGTACTCGCGGAGAGATGGAAGAGTATCGCCATAGTCGCCTTCAAGAGGGCGCTAAGGTATACGCTGAGCAGTTCGGCATCGACCCTGAGGACGTTGATTATCAGCGTGGTTTCAACGGGGACATTACCGAGCg                                                                                                                                                     <  1:47050/301‑1 (MQ=255)`  
                                                                                                                                                           `tCCGTAAGCTGACCCCTGAGCAACGTCGAGAAGCTCTCAACAACGGGACCCTTCTGTATCAGGATGACCCATACGCTATGGAAGCACTCCGAGTCAAGACTGGTCGTAACGCTGCGTATCTTGTGGACGATGACGTTATGCAGAGGATAAAAGAGGGTGTCTTCCGTACTCGCGAAGAGATGGAAGAGTATCGCCATAGTCGCCTTCAAGAGGGCGCTAAGGTATACGCTGAGCAGTTCGGCATCGACCCTGAGGACGTTGATTATCAGCGTGGTTTCAACGGGGACATTAcctgtctctt                                                                                                                                                  >  1:86863/1‑293 (MQ=255)`  
                                                                                                                                                                   `cTGACCCCTGAGCAACGTCGAGAAGCTCTCAACAACGGGACCCTTCTGTATCAGGATGACCCATACGCTATGGAAGCACTCCTAGTCAAGACTGGTCGTAACGCTGCGTATCTTGTGGACGATGACGTTATGCAGCAGATAAAAGAGGGTGTCTTCCGTACTCGCGAAGAGATGGAAGAGTATCGCCATAGTCGCCTTCAAGAGGGCGCTAAGGTATACGCTGAGCAGTTCGGCATCGACCCTGAGGACGTTGATTATCAGCGTGGTTTCAACGGGGACATTACCGAGCGTAACATCTCGc                                                                                                                                          <  1:312039/301‑1 (MQ=255)`  
                                                                                                                                                                   `aagaacccGGAGCAACGTCGAGAAGTTCTAAACAACGGGACCCTTATGTATCAGGATGACCCATACGCTATGGAAGCAATCCGGATCAAGACTGGTCGTAACGCTGCGTATCTTGTGGACGATGACGTTATGCAGCAGATAAAAGAGGGTGTCTTCCGTACTCGCGAAGAGATGGAAGAGTATCGCCATAGTCGCCTTCAAGAGGGCGCTAAGGTATACGCTGAGCAGTTCGGCATCGACCCTGAGGACGTTGATTATCAGCGTGGTTTCAACGGGGACATTACCGAGCGTAACATCTCGc                                                                                                                                          <  1:197745/296‑1 (MQ=255)`  
                                                                                                                                                                     `gACCCCTGAGCAACGTCGAGAAGCTCTCAACAACGGGACCCTTCTGTATCAGGATGACCCATACGCTATGGAAGCACTCCGAGTCAAGACTGGTCGTAACGCTGCGTACCTTGTGGACGATGACGTTATGCAGCAGATAAAAGAGGGTGTCTTCCGTACTCGCGAAGAGATGGAAGAGTATCGCCATAGTCGCCTTCAAGAGGGCGCTAAGGTATACGCTGAGCAGTTCGGCATCGACCCTGAGGACGTTGATTATCAGCGTGGTTTCAACGGGGACATTACCGAGCGTAACATCTCGCTg                                                                                                                                        >  1:26964/1‑301 (MQ=255)`  
                                                                                                                                                                          `gagacagaCGTCGAGAAGCTCTCAACAACGGGACCCTTCTGTATCAGGATGACCCATACGCTATGGAAGCACTCCGAGTCAAGACTGGTCGTAACGCTGCGTATCTTGTGGACGATGACGTTATGCAGAGGATAAAAGAGGGTGTCTTCCGTACTCGCGAAGAGATGGAAGAGTATCGCCATAGTCGCCTTCAAGAGGGCGCTAAGGTATACGCTGAGCAGTTCGGCATCGACCCTGAGGACGTTGATTATCAGCGTGGTTTCAACGGGGACATTACCGAGCGTAACATCTCGCTGTATgg                                                                                                                                   <  1:50682/294‑1 (MQ=255)`  
                                                                                                                                                                            `gAGCAACGTCGAGAAGCTCTCAACAACGGGACCCTTCTGTATCAGGATGACCCATACGCTATGGAAGCACTCCGAGTCAAGACTGGTCGTAACGCTGCGTATCTTGTGGACGATGACGTTATGCAGAGGATAAAAGAGGGTGTCTTCCGTACTAGCGAAGAGATGGAAGAGTATCGCCATAGTCGCCTTCAAGAGGGCGCTAAGGTATACGCTGAGCAGTTCGGCATCGACCCTGAGGACGTTGATTATCAGCGTGGTTTCAACGGGGACATTACCGAGCGTAACATCTCGCTGTATGGTg                                                                                                                                 <  1:307470/301‑1 (MQ=255)`  
                                                                                                                                                                            `gAGCAACGTCGAGAAGCTCTCAACAACGGGACCCTTCTGTATCAGGATGACCCATACGCTATGGAAGCACTCCGAGTCAAGACTGGTCGTAACGCTGCGTATCTTGTGGACGATGACGTTATGCAGAAGATAAAAGAGGGTGTCTTCCGTACTCGCGCAGAGATGGAAGAGTATCGCCATAGTCGCCTTCAAGAGGGCGCTAAGATATACGCTGAGCAGTTCGGCATCGAACCTGAGGACGTTGATTATCAGCGTGGTTTCAACGGGGACATTACCGAGCGTAACATCTCGCTGTATGGTg                                                                                                                                 >  1:183378/1‑301 (MQ=255)`  
                                                                                                                                                                                   `gTCGAGAAGCTCTCAACAACGGGACCCTTCTGTATCAGGATGACCCATACGCTATGGAAGCACTCCGAGTCAAGACTGGTCGTAACGCTGCGTATCTTGTGGACGATGACGTTATGCAGCAGATAAAATAGGGTGTCTTCCGTACTCGCGAAGAGATGGAAGAGTATCGCCATAGTCGCCTTCAAGAGGGCGCTAAGGTATACGCTGAGCAGTTCGGCATCGACCCTGAGGACGTTGATTATCAGCGTGGTTTCAACGGGGACATTACCGAGCGTAACATCTCGCTGTATGGTGCGCATGa                                                                                                                          >  1:236816/1‑301 (MQ=255)`  
                                                                                                                                                                                   `gTCGAGAAGCTCTCAACAACGGGACCCTTCTGTATCAGGATGACCCATACGCTATGGAAGCACTCCGAGTCAAGACTGGTCGTAACGCTGCGTATCTTGTGGACGATGACGTTATGCAGAGGATAAAAGAGGGTGTCTTCCGTACTCGCGAAGAGATGGAAGAGTATCGCCATAGTCGCCTTCAAGAGGGCGCTAAGGTATACGCTGAGCAGTTCGGCATCGACCCTGAGGACGTTGATTATCAGCGTGGTTTCAACGGGGACATTACCGAGCGTAACATCTCGCTGTCTgtctcttatac                                                                                                                          >  1:272675/1‑291 (MQ=255)`  
                                                                                                                                                                                         `aaGCTCTCAACAACGGGACCCTTCTGTATCAGGATGACCCATACGCTATGGAAGAACACCGAGTCAAGACTGGTCGTAACGCTGCGTATCTTGTGGACGATGACGTTATGCAGAGGATAAAAGAGGGTGTCTTCCGTACTCGCGAAGAGATGGAAGAGTATCGCCATAGTCGCCTTCAAGAGGGCGCTAAGGTATACGCTGAGCAGTTCGGCATCGACCCTGAGGACGTTGATTATCAGCGTGGTTTCAACTGGGACATTACCGAGCGTAACATCTCGCTGTATGGTGCGCATGATAActt                                                                                                                    <  1:177233/301‑1 (MQ=255)`  
                                                                                                                                                                                           `gCTCACAACAACGGGACCCTTCGGTATCAGGATGACCCATACGCTATGGAAGCACTCCGAGGCAAGACTGGTCGTAACGCTGCGTATCTTGTGGACGATGACGTTATGCAGCAGATAAAAGAGGGTGTCTTCCGTACTCGCGAAGAGATGGAAGAGTATCGCCATAGTCGCCTTCAAGAGGGCGCTAAGGTATACGCTGAGCAGTTCGGCATCGACCCTGAGGACGTTGATTATCAGCGTGGTTTCAACGGGGACATTACCGATCGTAACATCTCGCTGTATGGTGCGCATGATAActtct                                                                                                                  <  1:164035/301‑1 (MQ=255)`  
                                                                                                                                                                                                 `aaagagacagcccTTCTGTATCAGGATGACCCATACGCTATGGAAGCACTCCGAGTCAAGACTGGTCGTAACGCTGCGTATCTTGTGGACGATGACGTTATGCAGCAGATAAAAGAGGGTGTCTTCCGTACTCGCGAAGAGATGGAAGAGTATCGCCATAGTCGCCTTCAAGAGGGCGCTAAGGTATACGCTGAGCAGTTCGGCATCGACCCTGAGGACGTTGATTATCAGCGTGGTTTCAACGGGGACATTACCGAGCGTAACATCTCGCTGTATGGTGCGCATGATAACTTCTTGAGcc                                                                                                            <  1:299622/291‑1 (MQ=255)`  
                                                                                                                                                                                                   `caacGGGACCCTTCTGTATCAGGATGACCCATACGCTATGGAAGCACTCCGAGTCAAGACTGGTCGTAACGCTGCGTATCTTGTGGACGATGACGTTATGCAGAGGATAAAAGAGGGTGTCTTCCGTACTCGCGAAGAGATGGAAGAGTATCGCCATAGTCGCCTTCAAGAGGGCGCTAAGGTATACGCTGAGCAGTTCGGCATCGACCCTGAGGACGTTGATTATCAGCGTGGTTTCAACGGGGACATTACCGAGCGTAACATCTCGCTGTATGGTGCGCATGATAActtctgtctcttt                                                                                                          >  1:12530/1‑293 (MQ=255)`  
                                                                                                                                                                                                             `gggctcggagatgtgtataagagacagaTGGAAGCACTCCGAGTCAAGACTGGTCGTAACGCTGCGTATCTTGTGGACGATGACGTTATGCAGAGGATAAAAGAGGGTGTCTTCCGTACTCGCGAAGAGATGGAAGAGTATCGCCATAGTCGCCTTCAAGAGGGCGCTAAGGTATACGCTGAGCAGTTCGGCATCGACCCTGAGGACGTTGATTATCAGCGTGGTTTCAACGGGGACATTACCGAGCGTAACATCTCGCTGTATGGTGCGCATGATAACTTCTTGAGCCAGCAAGCTCAGa                                                                                                <  1:263345/274‑1 (MQ=255)`  
                                                                                                                                                                                                             `cTTCTGTATCAGGATGACCCATACGCTATGGAAGCACTCCGAGTCAAGACTGGTCGTAACGCTGCGTATCTTGTGGACGATGACGTTATGCAGAGGATAAAAGAGGGTGTCTTCCGTACTCGCGAAGAGATGGAAGAGTATCGCCATAGTCGCCTTCAAGAGGGCGCTAAGGTATACGCTGAGCAGTTCGGCATCGACCCTGAGGACGTTGATTATCAGCGTGGTTTCAACGGGGACATTACCGAGCGTAACATCTCGCTGTATGGTGCGCATGATAACTTCTTGAGCCAGCAAGCTCAGa                                                                                                >  1:114174/1‑301 (MQ=255)`  
                                                                                                                                                                                                               `ggggaaaaagaacagcccAAACGTTAAGGAAGGACCCCGAATCAAGAATGGGAGGAAAGCTTGCTAAGCTGTGGACGAAGAAGTTTAGCAAGAAAAAAAAGAGGGTGTCTTACGGACTCGGGGAGGGAAGGAAGAGTATCGCCATAGTCGCCTTCAAGAGGGCGCTAAGGTATACGCTGAGCAGTTCGGCATCGACCCTGAGGACGTTGATTATCAGCGTGGTTTCAACGGGGACATTACCGAGCGTAACATCTCGCTGTATGGTGCGCATGATAACTTCTTGAGCCAGCAAGCTCAGAAg                                                                                              <  1:38016/286‑1 (MQ=255)`  
                                                                                                                                                                                                                        `ggATGACCCATACGCTATGGAAGCACTCCGAGTCAAGACTGGTCGTAACGCTTCGTATCTTGTGGACGATGACGTTATGCAGAGGATAAAAGAGGGTGTCTTCCGTACTCGCGAAGAGATGGAAGAGTATCGCCATAGTCGCCTTCAAGAGGGCGCTAAGGTATACGCTGAGCAGTTCGGCATCGACCCTGAGGACGTTGATTATCAGCGTGGTTTCAACGGGGACATTACCGAGCGTAACATCTCGCTGTATGGTGCGCATGATAACTTCTTGAGCCAGCAAGCTCAGACGGGCGCTATc                                                                                     >  1:20396/1‑301 (MQ=255)`  
                                                                                                                                                                                                                            `gACCCATACGCTATGGAAGCACTCCGAGTCAAGACTGGTCGTAACGCTGCGTATCTTGTGGACGATGACGTTATGCAGCAGATAAAAGAGGGTGTCTTCCGTACTCGCGAAGAGATGGAAGAGTATCGCCATAGTCGCCTTCAAGAGGGCGCTAAGGTATACGCTGAGCAGTTCGGCATCGACCCTGAGGACGTTGATTATCAGCGTGGTTTCAACGGGGACATTACCGAGCGTAACATCTCGCTGTATGGTGCGCATGATAACTTCTTGAGCCAGCAAGCTCAGAAGGGCGCTATCATGa                                                                                 >  1:57174/1‑301 (MQ=255)`  
                                                                                                                                                                                                                              `ccctttcGCTATGAACGCACTCCGAGTCAAGACTGGTCGTAACGCTGCGTATCTTGTGGACGATGACGTTATGCAGAGGATAAAAGAGGGTGTCTTCCGTACTCGCGAAGAGATGGAAGAGTATCGCCATAGTCGCCTTCAAGAGGGCGCTAAGGGATACGCTGAGCAGTTCGGCATCGACCCTGAGGACGTTGATTATCAGCGTGGTTTCAACGGGGACATTACCGAGCGTAACATCTCGCTGTATGGTGCGCATGATAACTTCTTGAGCCAGCAAGCTCAGAAGGGCGCTATCATGAAc                                                                               <  1:74190/295‑1 (MQ=255)`  
                                                                                                                                                                                                                              `cccATACGCTAGGGAAGAACGCCGAGTTAAGACTGGTCGTAACGCGGCGTATCTTGGGGAAGATGACGTTATGCAGCAGATAAAAGAGGGTGTCTTCCGTACTCGCGAAGAGATGGAAGAGTATCGCCATAGTCGCCTTCAAGAGGGCGCTAAGGTATACGCTGAGCAGTTCGGCATCGACCCTGAGGACGTTGATTATCAGCGTGGTTTCAACGGGGAAATTACCGAGCGTAACATCTCGCTGTATGGTGCGCATGATAACTTCTTGAGCCAGCAAGCTCAGAAGGGCGCTATCATGAAc                                                                               <  1:332920/301‑1 (MQ=255)`  
                                                                                                                                                                                                                              `agacagcgagaTGGAAGGACGCCGAGTAAAGAATGGTCGTAACGCTGCGTATCTTGTGGACGATGACGTTATGAAGAAGATAAAAGAGGGTGTCTGCAGTACTCGCGAAGAGATGGAAGAGTATCGCCATAGTCGCCTTCAAGAGGGCGCTAAGGTAGACGCTGAGCAGTTCGGCATCGACCCTGAGGACGTTGATTATCAGCGTGGTTTCAACGGGGACAGTACCGAGCGTAACATCTCGCTGTATGGTGCGAATGATAACTTCTTGAGCCAGCAAGCTCAGAAGGGCGCTATCATGaaa                                                                               <  1:340953/291‑2 (MQ=255)`  
                                                                                                                                                                                                                                 `attcGCTAAGGAAGAACTCCGGATCAAGTCTGGTCGTAACGCTTCGTATCTTATGGACGATGACTTTATGCAGAGGATAAAAGAGGGTGTCTTCCGTACGCGCGAAGAGATTGAAGAGTATCGCCATAGTCGCCTTAAAGAAGGCGCTAAGGTATACGCTGAGCAGTTCGGCATCGACCCTGAGGACGTTGATTATCAGCGTGGTTTCAACGGGAACATTACCGAGCGTAACATATCGATGTATGGTGCGCATGATAACTTCTTGAGCCAGCAAGCTCAGAAGGGCGCTATCATGAACAGc                                                                            <  1:60411/298‑1 (MQ=255)`  
                                                                                                                                                                                                                                    `cGCTATGGAAGCACTCCGAGTCAAGACTGGTCGTAACGCTGCGTATCTTGTGGACGATGACGTTATGCAGCAGATAAAAGAGGGTGTCTTCCGTACTCGCGAAGAGATGGAAGAGTATCGCCATAGTCGCCTTCAAGAGGGCGCTAAGGTATACGCTGAGCAGTTCGGCATCGACCCTGAGGACGTTGATTATCAGCGTGGTTTCAACGGGGACATTACCGAGCGTAACATCTCGCTGTATGGTGCGCATGATAACTTCTTGAGCCAGCAAGCTCAGACGGGCGCTATCATGAACAGCCgg                                                                         >  1:216129/1‑300 (MQ=255)`  
                                                                                                                                                                                                                                      `cTATGGAAGCACTCCGAGTCAAGACTGGTCGTAACGCTGCGTATCTTGTGGACGATGACGTTATGCAGAGGATAAAAGAGGGTGTCTTCCGTACTCGCGAAGAGATGGAAGAGTATCGCCATAGTCGCCTTCAAGAGGGCGCTAAGGTATACGCTGAGCAGTTCGGCATCGACCCTGAGGACGTTGATTATAAGCGTGGTTTCAACGGGGACATTACCGAGCGTAACATCTCGCTGTATGGTGCGCATGATAACTTCTTGAGCCAGCAAGCTCAGAAGGGCGCTATCATGAACAGCCGAGt                                                                       >  1:200174/1‑301 (MQ=255)`  
                                                                                                                                                                                                                                       `tATGGAAGCACTCCGAGTCAAGACTGGTCGTAACGCTGCGTATCTTGTGGACGATGACGTTATGCAGAAGATAAAAGAGGGTGTCTTCCGTACTCGCGAAGAGATGGAAGAGTATCGCCATAGTCGCCTTCAAGAGGGCGCTAAGGTATACGCTGAGCAGTTCGGCATCGACCCTGAGGACGTTGATTATCAGCGTGGTTTCAACGGGGACATTACCGAGCGTAACATCTCGCTGTATGGTGCGCATGATAACTTCTTGAGCCAGCAAGCTCAGAAGGGCGCTATCATGACCAGCCGAGTg                                                                      >  1:223916/1‑301 (MQ=255)`  
                                                                                                                                                                                                                                                `ccTCCGAGTCAAGACTGGTCGTAACGCTGCGTATCTTGTGGACGATGACGTTATGCAGAAGATAAAAGAGGGTGTCTTCCGTACTCGCGAAGAGATGGAAGAGTATCGCCATAGTCGCCTTCAAGAGGGCGCTAAGGTATACGCTGAGCAGTTCGGCATCGACCCTGAGGACGTTGATTATCAGCGTGGTTTCAACGGGGACATTACCGAGCGTAACATCTCGCTGTATGGTGCGCATGATAACTTCTTGAGCCAGCAAGCTCAGAAGGGCGCTATCCTGAACAGCCGAGTGGAACTCAAc                                                             <  1:256993/300‑1 (MQ=255)`  
                                                                                                                                                                                                                                                  `cccGAGTCAAGACTGGTCGTAACGCTGCGTATCTTGTGGACGATGACGTTATGCAGAGGATAAAAGAGGGTGTCTTCCGTACTCGCGAAGAGATGGAAGAGTATCGCCATAGTCGCCTTCAAGAGGGCGCTAAGGTATACGCTGAGCAGTTCGGCATCGACCCTGAGGACGTTGATTATCAGCGTGGTTTCAACGGGGACATTACCGAGCGTAACATCTCGCTGTATGGTGCGCATGATAACTTCTTGAGCCAGCAAGCTCAGAAGGGCGCTATCATGAACAGCCGAGTGGAACTCAACgg                                                           <  1:344417/300‑1 (MQ=255)`  
                                                                                                                                                                                                                                                     `gAGTCAAGACTGGTCGTAACGCTGCGTATCTTGTGTACGATGACGTTATGCAGAAGATAAAAGAGGGTGTCTTCCGTACGCGCGAAGAGATGGAAGAGTATCGCCATAGTCGCCTTCAAGAGGGCGCTAAGGTATACGCTGATCAGTTCGGCATCGACCCTGAGGACGTTGATTATCAGCGTGGTTTCAACGGGGACATTACCGAGCGTAACATCTCGCTGTATGGTGCGCATGATAACTTCTTGAGCCAGCAAGCTCAGAAGGGCGCTATCATGAACAGCCGAGTGGAACTCAACGgtgt                                                        <  1:295573/301‑1 (MQ=255)`  
                                                                                                                                                                                                                                                      `aGTCAAGACTGGTCGTAACGCTGCGTATCTTGTGGACGATGACGTTATGCAGCAGATAAAAGAGGGTGTCTTCCGTACTCGCGAAGAGATGGAAGAGTATCGCCATAGTCGCCTTCAAGAGGGCGCTAAGGTATACGCTGAGCAGTTCGGCATCGACCCTGAGGACGTTGATTATCAGCGTGGTTTCAACGGGGACATTACCGAGCGTAACATCTCGCTGTATGGTGCGCATGATAACTTCTTGAGCCAGCAAGCTCAGAAGGGCGCTATCATGAACAGCCGAGTGGAACTCAACGGTGTc                                                       <  1:302044/301‑1 (MQ=255)`  
                                                                                                                                                                                                                                                      `aGTCAAGACTGGTCGTAACGCTGCGTATCTTGTGGACGATGACGTTATGCAGAGGATAAAAGAGGGTGTCTTCCGTACTCGCGAAGAGATGGAAGAGTATCGCCATAGTCGCCTTCAAGAGGGCGCTAAGGTATACGCTGAGCAGTTCGGCATCGACCCTGAGGACGTTGATTATCAGCGTGGTTTCAACGGGGACATTACCGAGCGTAACATCTCGCTGTATGGTGCGCATGATAACTTCTTGAGCCAGCAAGCTCAGAAGGGCGCTATCATGAACAGCCGAGTGGAACTCAACGGTGTc                                                       >  1:304343/1‑301 (MQ=255)`  
                                                                                                                                                                                                                                                               `tGGTCGTAACGCTGCGTATCTTGTGGACGATGACGTTATGCAGAAGATAAAAGAGGGTGTCTTCCGTACTCGCGAAGAGATGGAAGAGTATCGCCATAGTCGCCTTCAAGAGGGCGCTAAGGTATACGCTGAGCAGTTCGGCATCGACCCTGAGGACGTTGATTATCAGCGTGGTTTCAACGGGGACATTACCGAGCGTAACATCTCGCTGTATGGTGCGCATGATAACTTCTTGAGCCAGCAAGCTCAGAAGGGCGCTATCATGAACAGCCGAGTGGAACTCAACGGTGTCCTTCAAGAc                                              <  1:136417/301‑1 (MQ=255)`  
                                                                                                                                                                                                                                                               `tGGTCGTAACGCGGCGTATCTTGTGGACGATGACGTTATGCAGCAGATAAAAGAGGGTGTCTTCCGTACTCGCGAAGAGATGGAAGAGTATCGCCATAGTCGCCTTCAAGAGGGCGCTAAGGTATACGCTGAGCAGTTCGGCATCGACCCTGAGGACGTTGATGATCAGCGTGGTTTCAACGGGGACATTACCGAGCGTAACATCTCGCTGTATGGTGCGCATGATAACTTCTTGAGCCAGCAAGCTCAGAAGGGCGCTATCATGAACAGCCGAGTGGAACTCAACGGTGTCCTTCAAGAc                                              <  1:141093/301‑1 (MQ=255)`  
                                                                                                                                                                                                                                                               `gggTCGTAACGCTGCGTTTCTTGTGGACGATGACGTTATGCAGCAGAAAAAAGAGGGTGTCTTCCGTACTCGGGAAGGGAATGAAGAGTATCGCCATAGTCGCCTTCAAGAGGGCGCTAAGGTATACGCTGAGCAGTTCGGCATCGACCCTGAGGACGTTGATTAACAGCGTGGTATCAACGGGGACATTACCGAGCGTAACATCTCGCTGTATGGTGCGCATGATAACTTCTTGAGCCAGCAAGCTCAGAAGGGCGCTATCATGAACAGCCGAGTGGAACTCAACGGTGTCCTTCAAGAc                                              <  1:190043/300‑1 (MQ=255)`  
                                                                                                                                                                                                                                                                `ggTCGTAACGCTGCGTATCTTGTGGACGATGACGTTATGCAGAGGATAAAAGAGGGTGTCTTCCGTACTCGCGAAGAGATGGAAGAGTATCGCCATAGTCGCCTTCAAGAGGGCGCTAAGGGATACGCTGAGCAGTGCGGCATCGACCCTGAGGAAGGTGATTATCAGCGTGGTCTCAACGGGGACAGTAGCGAGCGTAACATCTCGCTGTATGGTGCGCATGATAACTTCTTGAAGCAGCAAGCTCAGAAGGGCGCTATCATGAACAGCCGAGTGGAACTCAACGGGGGCCTTGAAGAcc                                             >  1:254295/1‑301 (MQ=255)`  
                                                                                                                                                                                                                                                                 `gTCGTAACGCTGCGTAGCTTGTGGACGATGACGTTATGCAGAAGATAAAAGAGGGTGTCTTCCGTACTCGCGAAGAGATGGAAGAGTATCGCCATAGTCGCCTTCAAGAGGGCGCTAAGGTATACGCTGAGAAGTTCGGCATCGACCCTGAGGACGTTGATTATCAGCGTGGTTTCAACGGGGACATTACCGAGCGTAACATCTCGCTGTATGGTGCGCATGATAACTTCTTGAGCCAGCAAGCTCAGAAGGGCGCTATCATGAACAGCCGAGTGGAACTCAACGGTGTCCTTCAAGAccc                                            <  1:63375/301‑1 (MQ=255)`  
                                                                                                                                                                                                                                                                  `tCGTAACGCTGCGTATCTTGTGGACGATGACGTTATGCAGCAGATAAAAGAGGGTGTCTTCCGTACTCGCGAAGAGATGGAAGAGTATCGCCATAGTCGCCTTCAAGAGGGCGCTAAGGTATACGCTGAGCAGTTCGGCATCGACCCTGAGGACGTTGATTATCAGCGTGGTTTCAACGGGGACATTACCGAGCGTAACATCTCGCTGTATGGTGCGCATGATAACTTCTTGAGCCAGCAAGCTCAGAAGGGCGCTATCATGAACAGCCGAGTGGAACTCAACGGTGTCATTCAAGACCCt                                           >  1:329316/1‑301 (MQ=255)`  
                                                                                                                                                                                                                                                                     `tAACGCTGCGTATCTTGTGGACGATGACGTTATGCAGAAGATAAAAGAGGGTGTCTTCCGTACTCGCGAAGAGATGGAAGAGTATCGCCATAGTCGCCTTCAAGAGGGCGCTAAGGTATACGCTGAGCAGTTCGGCATCGACCCTGAGGACGTTGATTATCAGCGTGGTTTCAACGGGGACATTACCGAGCGTAACATCTCGCTGTATGGTGCGCATGATAACTTCTTGAGCCAGCAAGCTCAGAAGGGCGCTATCATGAACAGCCGAGTGGAACTCAACGGTGTCCTTCAAGACCCTGat                                        >  1:265602/1‑301 (MQ=255)`  
                                                                                                                                                                                                                                                                         `gCTGCGTATCTTGTGGACGATGACGTTATGCAGCAGATAAAAGAGGGTGTCTTCCGTACTCGCGAAGAGATGGAAGAGTATCGCCATAGTCGCCTTCAAGAGGGCGCTAAGGTATACGCTGAGCAGTTCGGCATCGACCCTGAGGACGTTGATTATCAGCGTGGTTTCAACGGGGACATTACCGAGCGTAACATCTCGCTGTATGGTGCGCATGATAACTTCTTGAGCCAGCAAGCTCAGAAGGGCGCTATCATGAACAGACGAGTGGAACTCAACGGTGTCCTTCAAGACCCTGATAtgc                                    <  1:67394/301‑1 (MQ=255)`  
                                                                                                                                                                                                                                                                           `tGCGTATCGTGTGGACGATGACGTTATGCAGAAGATAAAAGAGGGTGGCTTCCGTACTCGCGAAGGGATGGAAGAGTATCGCCATAGTCGCCTTCAAGAGGGCGCTAAGGTATACGCTGAGCAGTTCGGCATCGACCCTGAGGACGTTGATTATCAGCGTGGTTTCAACGGGGACATTACCGAGCGTAACATCTCGCTGTATGGTGCGCATGATAACTTCTTGAGCCAGCAAGCTCAGAAGGGCGCTATCATGAACAGCCGAGTGGAACTCAACGGTGTCCTTCAAGACCCTGATAtgctg                                  <  1:30953/301‑1 (MQ=255)`  
                                                                                                                                                                                                                                                                            `gCGTATCTTGTGGACGATGACGTTATGCAGCAGATAAAAGAGGGTGTCTTCCGTACTCGCGAAGAGATGGAAGAGTATCGCCATAGTCGCCTTCAAGAGGGCGCTAAGGTATACGCTGAGCAGTTCGGCATCGACCCTGAGGACGTTGATTATCAGCGTGGTTTCAACGGGGACATTACCGAGCGTAACATCTCGCTGTATGGTGCGCATGATAACTTCTTGAGCCAGCAAGCTCAGAAGGGCGCTATCATGAACAGCCGAGTGGAACTCAACGGTGTCCTTCAAGACCCTGATAtgctgc                                 >  1:117420/1‑301 (MQ=255)`  
                                                                                                                                                                                                                                                                                 `tCTTGTGGACGATGACGTTATGCAGAAGATAAAAGAGGGTGTCTTCCGTACTCGCGAAGAGATGGAAGAGTATCGCCATAGTCGCCTTCAAGAGGGCGCTAAGGTATACGCTGAGCAGTTCGGCATCGACCCTGAGGACGTTGATTATCAGCGTGGTTTCAACGGGGACATTACCGAGCGTAACATCTCGCTGTATGGTGCGCATGATAACTTCTTGAGCCAGCAAGCTCAGAAGGGCGCTATCCTGAACAGCCGAGTGGAACTCAACGGTGTCCTTCAAGACCCTGATATGCTGcgtcgt                            >  1:278834/1‑301 (MQ=255)`  
                                                                                                                                                                                                                                                                                  `cTTGTGGACGATGACGTTATGCAGAGGATAAAAGAGGGTGTCTTCCGTACTCGCGAAGAGATGGAAGAGTATCGCCATAGTCGCCTTCAAGAGGGCGCTAAGGTATACGCTGAGCAGTTCGGCATCGACACTGAGGACGTTGATTATCAGCGTGGTTTCAACGGGGACATTACCGAGCGTAACATCAAGATGTATGGTGCGCATGACAACTTCTTGAGCCAGCAAGCGCAGAAGGGCCCTATCATGAACAGCCGAGTGGAACTCAACGGTGTCCTTCAAGACCCTGATAtgcctgcctctt                           >  1:83712/1‑292 (MQ=255)`  
                                                                                                                                                                                                                                                                                       `ggACGATGACGTTATGCAGAAGATAAAAGAGGGTGTCTTCCGTACTCGCAAAGAGATGTAAGAGGATCGCCATAGTCGCCTTAAAGAGGGCGCTAAGGTATACGCTGAGCAGTTCGGCATCGACCCTGAGGACGTTGATTATCAGCATGGTTTCAACGGGGACATTACCGAGCGTAACATCTCGCTGTATGGTGCGCATGATAACTTCTTGAGCCAGCAAGCTCAGAAGGGCGCTATCATGAACAGCCGAGTGGAACTCAACGGTGTCCTTCAAGACCCTGATATGCTGCGTCGTCCAGAc                      <  1:120870/301‑1 (MQ=255)`  
                                                                                                                                                                                                                                                                                          `cGATGACGTTATGCAGAGGATAAAAGAGGGTGTCTTCCGTACTCGCGAAGAGATGGAAGAGTATAGCCATAGTCGCCTTCAAGAGGGCGCTAAGGTATACGCTGAGCAGTTCGGCATCGACCCTGAGGACGTTGATTATCAGCGTGGTTTCAACGGGGACATTACCGAGCGTAACATCTCGCTGTATGGTGCGCATGATAACTTCTTGAGCCAGCAAGCTCAGAAGGGCGCTATCATGAACAGCCGAGTGGAACTCAACGGTGTCCTTCAAGACCCTGATATGCTGcgtctgtctcttata                   >  1:319890/1‑290 (MQ=255)`  
                                                                                                                                                                                                                                                                                               `aCGTTATGCAGCAGATAAAAGAGGGTGTCTTCCGTACTCGCGAAGAGATGGAAGAGTATCGCCATAGTCGCCTTCAAGAGGGCGCTAAGGTATACGCTGAGCAGTTCGGCATCGACCCTGAGGACGTTGATTATCAGCGTGGTTTCAACGGGGACATTACCGAGCGTAACATCTCGCTGTATGGTGCGCATGATAACTTCTTGAGCCAGCAAGCTCAGAAGGGCGCTATCATGAACAGCCGAGTGGAACTCAACGGTGTCCTTCAAGACCCTGATATGCTGCGTCGTCCAGACTctgctgc              >  1:144770/1‑300 (MQ=255)`  
                                                                                                                                                                                                                                                                                               `aCGTTATGCAGCAGATAAAAGAGGGTGTCTTCCGTACTCGCGAAGAGATGGAAGAGTATCGCCATAGTCGCCTTCAAGAGGGCGCTAAGGTATACGCTGAGCAGTTCGGCATCGACCCTGAGGACGTTGATTATCAGCGTGGTTTCAACGGGGACATTACCGAGCGTAACATCTCGCTGTATGGTGCGCATGATAACTTCTTGAGCCAGCAAGCTCAGAAGGGCGCTATCATGAACAGCCGAGTGGAACTCAACGGTGTCCTTCAAGACCCTGATATGCTGCGTCGTCCAGACTCTGCTGa              >  1:108012/1‑301 (MQ=255)`  
                                                                                                                                                                                                                                                                                               `aCGTTATGCAGAGGATAAAAGAGGGTGTCTTCCGTACTCGCGAAGAGATGGAAGAGTATCGCCATAGTCGCCTTCAAGAGGGCGCTAAGGTATACGCTGAGCAGTTCGGCATCGACCCTGAGGACGTTGATTATCAGCGTGGTTTCAACGGGGACATTACCGAGCGTAACATCTCGCTGTATGGTGCGCATGATAACTTCTTGAGCCAGCAAGCTCAGAAGGGCGCTATCCTGAACAGCCGAGTGGAACTCAACGGTGTCCTTCAAGACCCTGATATGCTGCGTCGTCCAGACTctgcctg              >  1:251850/1‑298 (MQ=255)`  
                                                                                                                                                                                                                                                                                                `cGTTATGCAGCAGATAAAAGAGGGTGTCTTCCGTACTCGCGAAGAGATGGAAGAGTATCGCCATAGTCGCCTTCAAGAGGGCGCTAAGGTATACGCTGAGCAGTTCGGCATCGACCCTGAGGACGTTGATTATCAGCGTGGTTTCAACGGGGACATTACCGAGCGTAACATCTCGCTGTATGGTGCGCATGATAACTTCTTGAGCCAGCAAGCTCAGCAGGGCGCTATCATGAACATCCGAATGGAACTCAACGGTGTCCTTCAAGACCCTGATATGCTGCGTCGTCCAAACTCTGCTGAc             >  1:186176/1‑301 (MQ=255)`  
                                                                                                                                                                                                                                                                                                    `aTGCAGAGGATAAAAGAGGGTGTCTTCCGTACTCGCGAAGAGATGGAAGAGTATCGCCATAGTCGCCTTCAAGAGGGCGCTAAGGTATACGCTGAGCAGTTCGGCATCGACCCTGAGGACGTTGATTATCAGCGTGGTTTCAACGGGGACATTACCGAGCGTAACATCTCGCTGTATGGTGCGCATGATAACTTCTTGAGCCAGCAAGCTCAGAAGGGCGCTATCATGAACAGCCGAGTGGAACTCAACGGTGTCCTTCAAGACCCTGATATGCTGCGTCGTCCAGACTCTGCTGActtct         >  1:293101/1‑301 (MQ=255)`  
                                                                                                                                                                                                                                                                                                      `gCAGAGGATAAAAGAGGGTGTCTTCCGTACTCGCGAAGAGATGGAAGAGTATCGCCATAGTCGCCTTCAAGAGGGCGCTAAGGTATACGCTGAGCAGTTCGGCATCGACCCTGAGGACGTTGATTATCAGCGTGGTTTCAACGGGGACATTACCGAGCGTAACATCTCGCTGTATGGTGCGCATGATAACTTCTTGAGCCAGCAAGCTCAGAAGGGCGCTATCATGAACAGCCGAGTGGAACTCAACGGTGTCCTTCAAGACCCTGATATGCTGCTTCGTCCAGACTCTGCTGACTTCttt       >  1:43801/1‑301 (MQ=255)`  
                                                                                                                                                                                                                                                                                                         `gcagaTAAAAGAGGGTGTCTTCCGTACTCGCGAAGAGATGGAAGAGTATCGACATAGTCGCCTTCAAGAGGGCGCTAAGGTATACGCTGAGCAGTTCGGCATCGACCCTGAGGACGTTGATTATCAGCGTGGTTTCAACGGGGACATTACCGAGCGTAACATCTCGCTGTATGGTGCGCATGATAACTTCTTGAGCCAGCAAGCTCAGAAGGGCGCTATCATGAACAGCCGAGTGGAACTCAACGGTGTCCTTCAAGACCCTGATATGCTGCGCCGTCCAGACTCTGCTGACTTCTTTgag    >  1:208712/3‑301 (MQ=255)`  
                                                                                                                                                                                                                                                                                                         `gaagaTAAAAGAGGGTGTCTTCCGTACTCGCGAAGAGATGGAAGAGTATCGCCATAGTCGCCTTCAAGAGGGCGCTAAGGTATACGCTGAGCAGTTCGGCATCGACCCTGAGGACGTTGATTATCAGCGTGGTTTCAACGGGGACATTACCGAGCGTAACATCTCGCTGTATGGTGCGCATGATAACTTCTTGAGCCAGCAAGCTCAGAAGGGCGCTATCATGAACAGCCGAGTGGAACTCAACGGTGTCCTTCAAGACCCTGATATGCTGCGTCGTCCAGACTCTGCTGACTTCTTTgag    >  1:111555/1‑301 (MQ=255)`  
                                                                                                                                                                                                                                                                                                           `agaTAAAAGAGGGTGTCTTCCGTACTCGCGAAGAGATGGAAGAGTATCGCCATAGTCGCCGTCAAGAGGGCGCTAAGGTATACGCTGAGCAGTTCGGCATCGACCCTGAGGACGTTGATTATCAGCGTGGTTTCAACGGGGACATTACCGAGCGTAACATCTCGCTGTATGGTGCGCATGATAACTTCTTGAGCCAGCAAGCTCAGAAGGGCGCTATCATGAACAGCCGAGTGGAACTCAACGGTGTCCTTCAAGACCCTGATATGCTGCGTCGTCCAGACTCTGCTGACTTCTTTGAGaa  <  1:161340/301‑1 (MQ=255)`  

                                                                                                                                                                                                                                                                                                           `|`                                                                                                                                                                                                                                                                                                              
`AGGTATGGGCTATCGTGCAGCAACCACTCAGGCCGAACAGCCAAGGTCAAGCCTATTGGACACCATTGGTCGGTTCGCTAAGGCTGGTGCCGATATGTATACCGCTAAGGAACAACGAGCACGAGACCTAGCTGATGAACGCTCTAACGAGATTATCCGTAAGCTGACCCCTGAGCAACGTCGAGAAGCTCTCAACAACGGGACCCTTCTGTATCAGGATGACCCATACGCTATGGAAGCACTCCGAGTCAAGACTGGTCGTAACGCTGCGTATCTTGTGGACGATGACGTTATGCAGAAGATAAAAGAGGGTGTCTTCCGTACTCGCGAAGAGATGGAAGAGTATCGCCATAGTCGCCTTCAAGAGGGCGCTAAGGTATACGCTGAGCAGTTCGGCATCGACCCTGAGGACGTTGATTATCAGCGTGGTTTCAACGGGGACATTACCGAGCGTAACATCTCGCTGTATGGTGCGCATGATAACTTCTTGAGCCAGCAAGCTCAGAAGGGCGCTATCATGAACAGCCGAGTGGAACTCAACGGTGTCCTTCAAGACCCTGATATGCTGCGTCGTCCAGACTCTGCTGACTTCTTTGAGAA  >  NC_001604.1/28393‑28992`

  

Alignment Legend

Aligned base mismatch/match (shaded by quality score): `ATCG/ATCG` `< 3 ≤` `ATCG/ATCG` `< 9 ≤` `ATCG/ATCG` `< 21 ≤` `ATCG/ATCG` `< 34 ≤` `ATCG/ATCG` `< 38 ≤` `ATCG/ATCG`

Unaligned base: `atcg`    Masked matching base: `atcg`    Alignment gap: `‑`    Deleted base: `‑`
